# Combined Effects of Cyclooxygenase-1 and Cyclooxygenase-2 Selective Inhibitors on Ovarian Carcinoma *in Vivo*

**DOI:** 10.3390/ijms12010668

**Published:** 2011-01-18

**Authors:** Wei Li, Jie Wang, Hong-Ru Jiang, Xiao-Li Xu, Jun Zhang, Mei-Lin Liu, Ling-Yun Zhai

**Affiliations:** Department of Gynecology, Nanjing Medical University of Hangzhou Hospital, 261 Huansha Road, Hangzhou, Zhejiang 310006, China; E-Mails: hongsheqiao@sina.com (J.W.); hongrujiang@tom.com (H.-R.J.); joyce.xiaolixu@gmail.com (X.-L.X.); viola0116@163.com (J.Z.); lml13588722926@163.com (M.-L.L.); 494610422@qq.com (L.-Y.Z.)

**Keywords:** ovarian carcinoma, mice, cyclooxygenase selective inhibitor, cell proliferation, apoptosis

## Abstract

The present study was designed to investigate the combined effects of cyclooxygenase (COX)-1 and COX-2 selective inhibitors on human ovarian SKOV-3 carcinoma cells xenograft-bearing mice. The animals were treated with 3 mg/kg SC-560 (a COX-1 selective inhibitor) alone, 25 mg/kg celecoxib (a COX-2 selective inhibitor) alone, or SC-560/celecoxib by gavage, twice a day for three weeks. To test the mechanism of inhibition of tumor growth by COX selective inhibitors, the index of proliferating cells in tumor tissues was determined by immunostaining and the index of apoptotic cells by the terminal-deoxynucleotidyl-transferase-mediated deoxyuridine triphosphate nick end labeling (TUNEL) method. The inhibitory rate on tumor growth in the combination group was 35.54% which is significant statistically compared with that of the control group (*P* < 0.05). In the combination group, the index of cell proliferation and apoptosis were 12.40% and 51.03% respectively, which are significant statistically compared with those of the control group (22.56%, 19.07%, all *P* < 0.05). These studies indicate that synergism between two COX inhibitors and inhibitor combination treatment has particular potential for chemoprevention of ovarian cancer growth.

## 1. Introduction

Ovarian cancer represents the leading cause of death among gynecological malignancies. Despite recent advances in surgery and chemotherapy, improvement in long-term survival of these patients has been slight [1]. Chemical carcinogenesis experiments and epidemiological and clinical studies have collectively identified prostaglandins and their rate-limiting enzymes, cyclooxygenase (COX)-1 and COX-2, as molecules involved in the onset and progression of a variety of malignancies [2]. Research primarily focusing on colorectal cancer has provided strong evidence that nonsteroidal anti-inflammatory steroids (NSAID) are effective in both cancer prevention and treatment of established tumors [3]. NSAIDs block arachidonic acid metabolism by inhibiting COX, the enzyme that catalyze the rate-limiting step, and thus reducing levels of prostaglandins. Two enzyme isoforms of COX are known, referred to as COX-1 and COX-2, they are encoded by separate genes, and they have very similar structural and kinetic properties and show distinct cell-specific expression and regulation [4]. COX-1 is constitutively expressed in most tissues and plays a role in various physiologic functions, whereas COX-2 is transiently inducible by stimuli such as cytokines, growth factors, mitogens, tumor promoters and hormones and also regulates inflammation, differentiation, mitogenesis, and angiogenesis [4–7].

Recently, a concerted effort has been focused on COX-2 selective inhibitors since COX-2 expression is a characteristic feature of most malignant neoplasms. Research primarily has demonstrated that COX-2 is up-regulated in a range of cancers, particularly colorectal cancer [3], but also gastric [5], breast [8], thyroid [9], and ovarian cancers [10–12]. Moreover, elevated COX-2 expression has been identified as an independent prognostic factor [10] that is associated with reduced survival [11] and poor response to standard combination chemotherapy in ovarian cancer [12]. By using COX-2 selective inhibitors featuring disruption of the gene encoding this enzyme, relevance to carcinogenesis in various organs, including the ovarian, has been shown [6,13,14]. Possible involvement of COX-1 in ovarian cancer has also been reported. Daikoku T. *et al.* [15] found COX-1 to be the predominant COX isoforms expressed in ovarian cancer. Studies have shown that overexpression of COX-1 is associated with elevated levels of angiogenic factors in ovarian carcinoma, which was inhibited by COX-1 selective inhibitors [16]. These results indicate that the COX participates in the progression of ovarian carcinoma and could be targeted for anti-tumor therapy.

NSAIDs are thought to impede cancer growth primarily by attenuating COX activity, although other non-COX targets can not be ruled out. The effects of COX-1 selective inhibitors in attenuating tumor growth *in vivo* are remarkable [15], whereas, COX-2 selective inhibitors have potent antineoplastic effects *in vivo* in preclinical models of various solid malignancies [3,10]. These findings led to the initiation of a number of animal and clinical trials examining the efficacy of COX-1 and COX-2 selective inhibitors in primary and/or secondary prevention of cancer or as part of a combination therapy regimen for established tumors [3,17]. Many, but not all studies show that long-term use of NSAIDs reduces the risk of cancer [17,18]. Regular use of ibuprofen or aspirin decreased breast cancer rates by about 50% and 40%, respectively [18]. It appears that NSAIDs use significantly protects against some but not all types of human cancer. However, epidemiologic studies examining whether NSAIDs can prevent or delay the development of ovarian cancers remain inconclusive.

In the present study, we postulate that a combination of COX-1 and COX-2 selective inhibitors should reduce the growth of tumors more effectively than either agent alone in nude female mice transplanted with a human ovarian cancer SKOV-3 cell line. To test this possibility, combined effects of SC-560, a COX-1 selective inhibitor, and celecoxib, a COX-2 selective inhibitor, on ovarian tumor growth *in vivo* were examined. In addition, we also examined the anti-tumor mechanisms by which SC-560 and celecoxib affects ovarian cancer tumor growth.

## 2. Results and Discussion

### 2.1. Inhibition of Ovarian Cancer Growth

**Figure 1:**
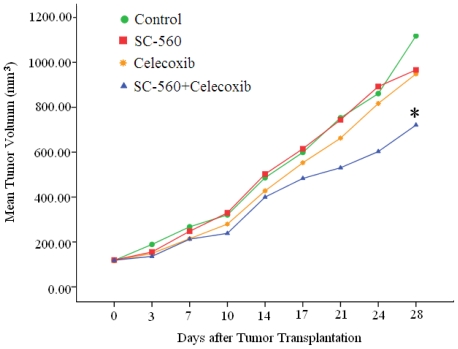
Effects of SC-560 and celecoxib on tumor growth *in vivo*. The inhibitory of SC-560 and celecoxib on tumor growth were determined in an ovarian cancer model using SKOV-3 cells. After 7 days to allow tumor establishment, mice were treated with SC-560 and celecoxib. Treatment was continued for 21 days. Average tumor volume in SC-560 and celecoxib combination group was significantly different from vehicle-treated mice at day 28. Statistical significance was determined using Student’s *t*-test. * *P* < 0.05.

To test whether SC-560 or celecoxib could inhibit ovarian cancer growth, we used the human ovarian carcinoma cell line SKOV-3. SKOV-3 cells were implanted into the subcutaneous growth, so that changes in tumor growth could be easily monitored. The tumor growth increased throughout the period examined in the control group whereas the growth was substantially suppressed in the combination group. Data in Figure 1 show the relative effect of SC-560 or/and celecoxib therapy. SC-560 or celecoxib administrated alone by gavage twice every other day for 21 consecutive days at 3 mg/kg body weight, 25 mg/kg body weight respectively did not prevent the growth of ovarian carcinoma, but they both showed a decreasing tendency in growth-inhibitory effect compared with the control group. For example, after three weeks of treatment with SC-560, a mean tumor volume of 966 mm^3^ was observed on day 28. Under similar conditions, celecoxib-treated animals showed a mean tumor volume of 948 mm^3^, whereas mean tumor volume in control mice was 1118 mm^3^. The effects of SC-560 combined with celecoxib in attenuating tumor growth are remarkable during the entire treatment period. On day 28, tumor volume in the combination group was reduced by 35.54% compared with control mice; the inhibitory effect of the combination group is significant statistically compared with that of control group (*P* < 0.05). These results suggest that the combination of COX-1 and COX-2 selective inhibitors may have chemopreventive properties on ovarian cancer.

### 2.2. Synergistic Effects of SC-560 and Celecoxib on Ovarian Cancer Growth

SC-560 and celecoxib alone showed inhibition of tumor growth by 13.57% and 15.16%, respectively, in this experiment. However, a combination of SC-560 and celecoxib showed better antitumor activity with about 35.54% inhibition of tumor growth. Table 1 summarizes relative tumor volume of control and treated groups at three different time points. Combination therapy showed more than additive effect on tumor growth inhibition. On day 17, there was 1.2-fold improvement in antitumor activity in the combination group when compared with the expected additive effect. At this time point, celecoxib alone inhibited tumor growth by 8% (fractional tumor volume, 0.923 mm^3^) when compared with the control group. With time, there was a progressive improvement in antitumor activity. On day 24, SC-560 and celecoxib combination group showed a 1.4-fold higher inhibition of tumor growth over additive effect (expected fractional tumor volume).

### 2.3. COX Expression in Ovarian Carcinoma Cells

**Figure 2:**
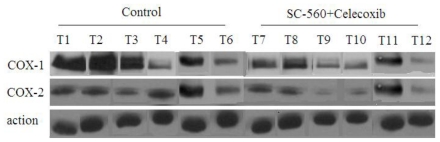
COX protein levels in xenograft tumors of nude mice treated or not treated with combined treatment of SC-560 and celecoxib. COX-1 and COX-2 protein levels were analyzed by Western blotting. Anti-β-actin was used as a control for equal loading. Lanes 1–6: tumor tissues of six mice in control group. Lanes 7–12: tumor tissues of six mice in the combination group.

**Figure 3:**
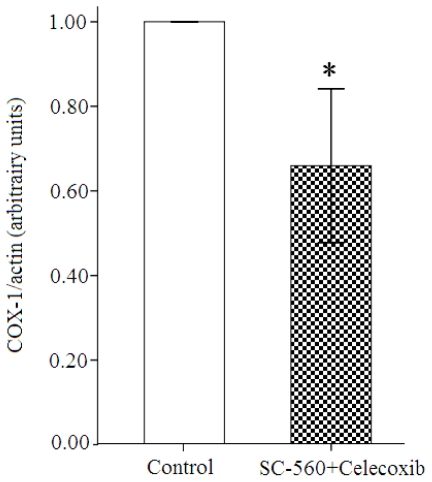
The ratios of COX-1/actin after autoradiography integrations. Results were expressed in arbitrary units. The COX-1 expression was decreased significantly by 31% in the combination group compared with the control group (* *P* < 0.01; error bars indicate SE).

To investigate whether the COX inhibitors can regulate COX-1 or COX-2 expression in ovarian carcinoma, the combination group and control group were analyzed for expression of both COXs expression by Western blotting. Western blotting analyses of COXs expression show that the levels of COX-1 and COX-2 protein expression are reduced in the combination group compared with the control group (Figure 2). Moreover, the levels of COX-1 protein expression are substantially reduced in the combination group compared with the control group (31% reduction, *P* < 0.01, Figure 3). Whereas the levels of COX-2 protein in the combination group only revealed a 24% diminution compared to the control group.

### 2.4. Cell Proliferation

**Figure 4:**
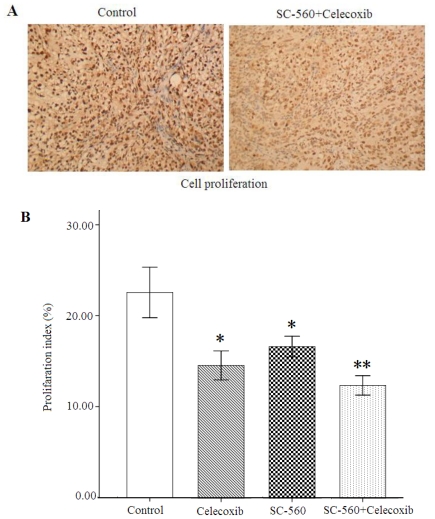
Cell proliferation in xenograft tumors of nude mice treated or not treated with SC-560 and/or celecoxib. (**A**) Immunostaining of cell proliferation (Ki-67) by immunohistochemistry. The combination group of COX selective inhibitor SC-560 and celecoxib attenuates tumor cell proliferation. (**B**) The index of cell proliferation was determined from the ratio of nuclear Ki-67 protein-positive cells/total nuclei number by immunohistochemical method. * *P* < 0.05, ** *P* < 0.01, compared with control; error bars indicate SE.

We employed cell growth in allografted tumors in nude mice treated with vehicle SC-560, celecoxib or SC-560/celecoxib, assessed by proliferation-associated nuclear antigen (Ki-67) staining. As shown in Figure 4A, the population of Ki-67-positive cells in tumor sections was substantially lower when the mice were exposed to SC-560/celecoxib than in those receiving the vehicle. Data for the proliferation index of four groups are shown in Figure 4B. In SC-560 or celecoxib alone group, the proliferation index were 16.67 ± 3.13% or 12.40 ± 2.92%, which are significant statistically compared with that of the control group (22.56 ± 7.62%, both *P* < 0.05). Furthermore, the combination group showed a 45.04% reduction of the proliferation index (12.40 ± 2.92%) at the end of treatment compared with the control group (*P* < 0.01).

### 2.5. Cell Apoptosis

**Figure 5:**
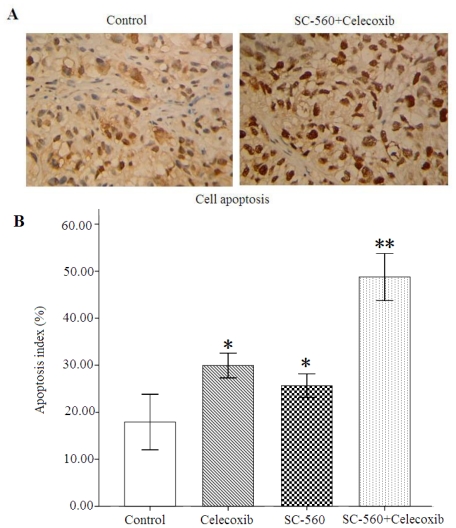
Cell apoptosis in xenograft tumors of nude mice in either the presence or absence of SC-560 and/or celecoxib. (**A**) Immunostaining of cell apoptosis in tumors by TUNEL. The combination group of COX selective inhibitor SC-560 and celecoxib accelerates tumor cell apoptosis. (**B**) The index of cell apoptosis was determined from the ratio of nuclear apoptosis-positive cells/total nuclei number. * *P* < 0.05, ** *P* < 0.01, compared with control; error bars indicate SE.

We also employed cell apoptosis in these four groups, assessed by TUNEL. As shown in Figure 5A, the number of apoptotic cells was more frequent in tumor sections of the combination group than in those of the control group. Data for the apoptosis index of four groups are shown in Figure 5B. The apoptosis index was 25.63 ± 7.58% or 29.94 ± 7.88% in SC-560 or celecoxib alone group, which is significant statistically compared with that of the control group (19.07 ± 16.36%, both *P* < 0.05). In addition, the combination group showed a 167.6% increase of the apoptosis index (51.03 ± 14.75%) at the end the treatment compared with the control group (*P* < 0.01).

### 2.6. Discussion

The present study was conducted to assess how tumor development is modified by COX selective inhibitors. This study demonstrates that SC-560 and celecoxib, when administered together, resulted in a synergistic anti-tumor effect when compared with treatment with the same doses of either SC-560 or celecoxib alone, which highly significantly suppressed tumor growth compared with the control group on day 28. The effects of SC-560 combined with celecoxib in attenuating tumor growth *in vivo* proved remarkable during the entire treatment period. These results demonstrate that the combination of the two drugs enhances the antitumor activity against ovarian tumor. We have known in several previous studies that, indeed, these combination protocols can provide greater efficacy than the individual agents administered alone [19,20]. It is anticipated that the administration of a combination of chemopreventive agents, which are selected based on definitive mechanisms relevant to tumorigenesis, should have beneficial applications in human cancer chemoprevention trials. Likewise, COX inhibition procedures are now being examined for the preclinical treatment of tumor [15,16]. Inhibitors of COX-1 are being shown to be more effective in improving tumor responsiveness when combined with other agents [21]. Whereas Shipeng Z. *et al.* [22] found that celecoxib could inhibit tumor growth and enhance the antitumor effects of oxaliplatin through their synergistic role in inhibiting different targets *in vivo*. Our results strongly support the consideration and development of protocols to evaluate the preclinical efficacy of combining COX-1 inhibitor with COX-2 inhibitor therapy. In the present study, both COX inhibitors were given together in a fixed schedule and dose. Therefore, the observed synergism can be further improved by modulating dosage and frequency of administration based on pharmacokinetics, distribution, and bioavailability.

Epidemiological and clinical studies have collectively identified COX-1 and COX-2 as molecules involved in the onset and progression of a variety of malignancies [2]. COX-1 and COX-2 inhibitors were reported to suppress tumor growth and metastasis in mice with established metastatic mammary tumors [23] and intestinal carcinoma [19]. Moreover, selective or nonselective COX inhibitors suppressed tumor growth and metastasis in mice with established epithelial ovarian cancer [24], modulating tumor angiogenesis improved survival of mice in mouse model of colorectal cancer [25]. It was reported that in *APC* (*adenomatous polyposis coli*) mice, which serve as a genetically defined model of FAP (familial adenomatous polyposis), the combination treatment with COX-1 and COX-2 selective inhibitors more effectively suppressed polyp growth than either of the single treatments alone [19]. Additional evidence that a combination therapy approach using a COX-1 and a COX inhibitor was more effective than either procedure alone at producing short-term tumor cures is shown in [20]. Li S. *et al*. [26] found that COX-1 and COX-2 are expressed in every type of ovarian epithelial cancer. Thus, both COXs may contribute to tumorigenesis in various organs.

In this study, in comparison to the combination group, SC-560 or celecoxib alone led to less tumor inhibition during the entire treatment period, only showing decreasing tendency in growth-inhibitory effect of human ovarian SKOV-3 tumors compared to those with the control. This result differed from previous studies which reported administered COX-1 and COX-2 selective inhibitor alone could highly significantly decrease tumor growth [6,15]. The discrepancies may depend on difference in dosage and frequency of administration.

Unrestricted cell proliferation and reduced apoptosis are hallmarks of transformed cells. A plethora of signaling pathways and molecules influences these processes. Our results of reduced tumor growth with decreased cell proliferation and accelerated apoptosis following the combination treatment suggest that COXs inhibitors suppress ovarian tumor growth at least by influencing cell proliferation and apoptosis. Inhibition of cell proliferation and the induction of apoptosis are believed to be responsible, in part, for the chemo-preventative effects of NSAIDs illustrated in many reviews [15,27,28]. It was reported that SC-560 and celecoxib were both effective at inhibiting the growth of COX-deficient HCT-15 colon cancer xenograft in nude mice and induced apoptosis *in vitro* [29]. Furthermore, Frank G. *et al*. [28] found that the SC-560 induce apoptosis and inhibit tumor growth *in vivo*, and one mechanism may involve changes in gene expression by COX inhibitors which is dependent on the structural character of the COX selective inhibitor rather than its ability to selectively inhibit COX-1 and COX-2. This is in agreement with Zhu *et al*. who reported that NSAIDs induce apoptosis independent of their ability to inhibit COX [30]. Therefore, both COX-dependent and independent mechanisms are probably involved in the chemo-preventative activity of these compounds.

The COX isoforms possibly compensate for lack of expression of the other [31]. Dual-COX inhibition would be expected to overcome such compensation in ovarian tumorigenesis and this may be one of the reasons for the combination effect. However, most traditional COX inhibitors, such as indomethacin are more potent inhibitors of COX-1 than COX-2. Recently, a majority of tumors overexpresses COX-2 and not COX-1; this is consistent with the hypothesis that COX-1 is constitutively expressed and responsible for basal, whereas COX-2 is highly inducible and responsible for pro-inflammatory cytokines and growth factors, both of which are likely to be highly concentrated within the microenvironment of a tumor. The basis for the divergent expression patterns of COX-1 and COX-2 in ovarian cancer is not known. In this experiment, we observed that both COXs protein levels were reduced in the combination group cells compared with those in the control group, but the levels of COX-1 proteins were markedly inhibited by SC-560 combined with celecoxib in tumor cells, which suggests that oncogenic transformation leads to the expression of COX-1 in the ovarian tissue. Similar results were obtained by Daikoku T. *et al.* [15,24]. Moreover, Hales D.B. *et al.* [32] and Urick M.E. *et al.* [33] both found high expression of COX-1, not COX-2, in ovarian cancer in the domestic hen (Gallus domesticus); others confirmed COX-1 expression but not COX-2 expression in SKOV-3 tumors [34]. Dore M. *et al*. [35] used immunohistochemistry to demonstrate strong expression of COX-1, not COX-2, protein in human ovarian cancer specimens. It was reported that COX-1 expression regulates angiogenesis in endothelial cell [36]. Induced overexpression of COX-1 in endothelial cells leads to malignant transformation [37]. These findings suggest COX-1 may be the predominant pathway compared with COX-2 in the development of ovarian cancer.

## 3. Experimental Section

### 3.1. Human Ovarian Tumors in Nude Mice

SKOV-3 cells were used for tumor growth studies *in vivo*. SKOV-3 was purchased from China Type Culture Collection and grown in the recommended media under standard conditions. SKOV-3 cells were implanted sub cutaneously in the dorsal skin (2 × 10^6^ cells) of female athymic nude mice (nu/nu, 7–8 weeks old). When the tumors became visible (7 days after inoculation), mice were randomly separated into four groups (six mice in each group): SC-560, celecoxib, SC-560/celecoxib and control.

COX inhibitors, SC-560 (Sigma Chemical Co. St. Louis, MO, U.S.) and Celecoxib (Pfizer Co. Groton, CT, U.S.) were administered by gavage in a 0.5 ml suspension of 0.5% methylcellulose (Sigma Chemical Co. St. Louis, MO, U.S.) 0.025% Tween 20 (Sigma Chemical Co.) at a dose of 3 mg/kg (SC-560), 25 mg/kg (celecoxib) twice a day. The dose was chosen for their specificity in inhibiting COX isotypes [38]. SC-560 alone, celecoxib alone, or SC-560 in combination with celecoxib was each given by gavage twice every other day. A control group of mice was treated with sterile PBS under similar conditions. Drugs or vehicle were administered for a period of 21 days, beginning on the day one week after the tumors became palpable.

The tumor dimensions were measured twice a week using a linear caliper, and tumor volume was calculated using the equation *V* (mm^3^) = *a* × *b*^2^/2, where *a* is the largest diameter and b is the smallest diameter [39]. Tumor growth was evaluated by the inhibition rate as assessed by the formula: *IR* = *C* – *T*/*C* × 100%. Where *IR* is the mean inhibition rate, *T* is the mean tumor volume in the treatment group, and *C* is the mean tumor volume in the control group. The animals were weighed weekly throughout the experiment. On day 28, all of the mice were sacrificed, and tumor tissue samples were collected and then fixed in 10% phosphate-buffered formalin solution for immunohistology or stored at –80 °C until analyzed. The tumor tissue samples were snap-frozen in liquid nitrogen before their storage at –80 °C.

### 3.2. Western Blot Analysis

Lysates (40 μg of protein/lane) were analyzed by SDS-PAGE on 12% Tris-glycine gels. Protein was electrotransferred to nitrocellulose membranes and blocked with a solution of PBS containing 5% milk and 0.1% Tween 20. Bands were detected using chemiluminescent detection reagents (GE healthcare, code: RPN2106). Blots were probed with a goat polyclonal antibody against COX-1 (Beijing biosynthesis biotechnology Co., China, code: bs-0582R), COX-2 (Beijing biosynthesis biotechnology Co., China, code: bs-0732R) followed by a peroxidase-conjugated antigoat (abcam), respectively. After incubation, antibodies were washed in PBS and 0.1% Tween 20. Bands were detected using chemiluminescent detection reagents (GE healthcare, code: RPN2106).

### 3.3. Immunohistochemistry

Immunobiochemical and molecular biologic characterization of the cell proliferation-associated nuclear antigen is defined by monoclonal antibody Ki-67. Immunostaining with monoclonal antibody Ki-67 provides a reliable means of rapidly evaluating the growth fraction of normal and neoplastic human or animal cell populations. To detect the Ki-67 nuclear antigen, which is present throughout the cell cycle, but absent in the dormant G_0_ phase [40], tumors were fixed in 10% neutral buffered formalin for 24–48 h prior before being embedded in paraffin. After deparaffinization, the tissue sections were heated at 121 °C for 15 min in 10 mM TrisHCl with 1 mM EDTA (pH 9.0). Endogenous peroxidase was blocked with 3% hydrogen peroxide in methanol for 10 min at room temperature. The samples were incubated with anti-Ki-67 antigen, clone MIB-5 (M7248), for 90 min at room temperature. Then, the sections were incubated in EnVision reagent for 40 min and DAB/H_2_O_2_ for 8–12 min at room temperature. Proliferation was assessed by counting the number of Ki-67 positively staining nuclei and total number of cancer cells at ×400 magnification in five representative regions of the tumor. The proliferation index was calculated as follows: proliferation index = (number of cells labeled with Ki-67/total cell number) × 100%.

### 3.4. TUNEL Assay

Apoptosis can be measured in the terminal transferase uridyl nick end labeling (TUNEL) assay by the TUNEL kit (Chemicon Co. Beijing zhongshan, China). TUNEL assay allows the easy demonstration of cell death as a result of apoptosis. The tissue samples were fixed in 4% paraformaldehyde for 24 h, dehydrated, and embedded in paraffin in the conventional manner. The paraffin-embedded tissues were cut into 4-lm-thick sections. After deparaffinization in a graded alcohol series, the tissue sections were covered with 20 μg proteinase K/mL PBS(–) for 15 min at room temperature, followed by blocking of endogenous peroxidase activity. The samples were then incubated with TdT enzyme and biotin-16-dUTP in TdT buffer containing 0.01% bovine serum albumin for 1.5 h at 37 °C in a humidity chamber. Biotin-16-dUTP nucleotides that had been incorporated into DNA fragments were detected using the ABC method with DAB as the chromogen. In each tissue specimen, five high-power fields (×400) were randomly selected; the apoptotic index was calculated in these fields as the percentage of positive cells, given by the following equation: apoptotic index = (number of positive cells/total number of cells) × 100% [41].

### 3.5. Statistical Analyses

Statistical analysis was performed with SPSS software (SPSS Standard version 17.0, SPSS). Statistical significance between control and treated groups was determined by Student’s *t*-test. All the experimental data were expressed as means values ± SE. Results were considered statistically significant when *P* values < 0.05.

## 4. Conclusions

The main finding of this study is that a combination therapy approach using SC-560 and celecoxib was more effective than either procedure alone on human ovarian SKOV-3 carcinoma cells xenograft-bearing mice at producing short-term tumor cures. The data showed that the combination use of SC-560 and celecoxib suppressed tumor growth at least by inhibiting cell proliferation and increasing apoptosis. Our results also indicate that, COX-1 may be the predominant pathway compared with COX-2 on ovarian cancer in the effect of combination use of COX-1 and COX-2 selective inhibitors. These observations support the hypothesis that a combination of COX-1 and COX-2 selective inhibitors may have better chemopreventive properties on ovarian cancer than when administered alone.

## Figures and Tables

**Table 1:** Combination therapy with SC-560 and Celecoxib.

Day b	Fractional Tumor Volume (FTV) Relative to Untreated Controls a				
	SC-560	Celecoxib	Combination Treatment		Ratio of Expected TV/Observed FTV d
			Expected c	Observed	
17	1.027	0.923	0.948	0.807	1.175
21	0.988	0.880	0.869	0.704	1.234
24	1.036	0.949	0.983	0.700	1.404

a FTV (mean tumor volume experimental)/(mean tumor volume control).

b Day after tumor cell transplantation.

c (Mean FTV of SC-560) × (mean FTV of Celecoxib).

d Obtained by dividing the expected FTV by the observed FTV. A ratio of >1 indicates a synergistic effect, and a ratio of <1 indicates a less than additive effect.
